# Malaria in the Americas: Trends from 1959 to 2011

**DOI:** 10.4269/ajtmh.14-0368

**Published:** 2015-02-04

**Authors:** Keith H. Carter, Prabhjot Singh, Oscar J. Mujica, Rainier P. Escalada, Maria Paz Ade, Luis Gerardo Castellanos, Marcos A. Espinal

**Affiliations:** Department of Communicable Diseases and Health Analysis, Pan American Health Organization/World Health Organization, Washington, DC; Special Program for Sustainable Development and Health Equity, Pan American Health Organization/World Health Organization, Washington, DC

## Abstract

Malaria has declined in recent years in countries of the American continents. In 2011, 12 of 21 endemic countries had already met their 2015 Millennium Development Goal. However, this declining trend has not been adequately evaluated. An analysis of the number of cases per 100,000 people (annual parasite index [API]) and the percentage of positive blood slides (slide positivity rate [SPR]) during the period of 1959–2011 in 21 endemic countries was done using the joinpoint regression methodology. During 1960–1979, API and SPR increased significantly and peaked in the 1980s. Since the 1990s, there have been significant declining trends in both API and SPR. Additionally, both *Plasmodium vivax* and *P. falciparum* species-specific incidence have declined. With the exception of two countries, such a collectively declining malaria trend was not observed in previous decades. This presents a unique opportunity for the Americas to seriously consider malaria elimination as a final goal.

## Introduction

Malaria, a global health problem and one of the reasons for the establishment of the Pan American Health Organization (PAHO) in 1902, affects millions of people and has been associated with approximately 1 million deaths each year.^1^,^2^ The estimated disability-adjusted life years (DALYs) lost because of malaria was 111,000 DALYs in the Latin American countries.^3^ In the past decade, the burden of disease has been decreasing on the North and South American continents. At the end of the year 2011, 12 of 21 presently malaria-endemic countries in the continents had already met their 2015 Millennium Development Goal (MDG; a 75% reduction in malaria incidence compared with the year 2000), whereas another 5 countries were well on the way to achieve it.^2^ Six of the endemic countries transitioned into the pre-elimination phase in 2011, having been so classified by the World Health Organization (WHO), and a number of other countries are making similar progress toward the achievement of the pre-elimination criteria.^2^

PAHO adopted continental malaria eradication (now referred to as regional malaria elimination) as a Regional Program in 1954, a year before the Global Malaria Eradication Program (GMEP) was launched in Mexico in 1955; during its implementation, many previously endemic countries achieved malaria elimination.^4^,^5^ Increasing parasite resistance to the antimalarial drug chloroquine and vector resistance to insecticides coupled with waning focus on combatting malaria led to an increase in malaria morbidity and mortality globally in the 1970s and 1980s.^6^ The burden and need for continued focus on the disease led to the adoption of the Global Malaria Control Strategy in 1992 at a Ministerial Conference in Amsterdam.^7^ The magnitude of the disease as well as its high mortality among children in Africa resulted in a call to counter malaria with the formation of the Roll Back Malaria partnership between the WHO, the United Nations Development Program (UNDP), and the World Bank in 1998. The initiative was adopted in the Americas in 2000.^8^ The United Nations General Assembly declared 2001–2010 as the decade to roll back malaria, and in 2012, they called for accelerated efforts to control and eliminate malaria.^9^,^10^ To support the achievement of these global goals and facilitate the achievement of country targets, PAHO coordinated the multistakeholder development and implementation of the Regional Strategy for Malaria in the Americas 2006–2010 and the Strategy and Plan of Action for Malaria 2011–2015.^11^,^12^

The history of malaria throughout the last half century in the Americas has been documented, but temporal trends have not been adequately evaluated.^5^,^13^ Studies have assessed the impact of renewed efforts for malaria control over the last decade.^14^ Nevertheless, doubt remains, because a formal and systemic analysis of malaria trends in countries has not been done under claims of data paucity or unreliability. This study makes use of more than five decades of yearly incidence data for malaria-endemic countries in the Americas and systemically analyzes them to identify temporal trends and detect years of significant change (i.e., joinpoints or change points) in their trajectories over time.

## Materials and Methods

Countries of the American continents annually report incident malaria cases to PAHO/WHO. These official country reports were used to compile annual data series for 21 endemic countries in the Americas from 1959 to 2011. Total number of slides examined, total confirmed cases, and cases by species type were analyzed. United Nations' mid-year annual population estimates for the corresponding year were used for standardization and allow comparison across time and countries.^15^

Annual data series for all countries were compiled. Analysis was done for four variables: annual parasite index (API) or the number of malaria cases per 100,000 inhabitants, slide positivity rate (SPR) or the number of cases per 100 blood slides examined, and *Plasmodium falciparum* and *P. vivax* rates or numbers of species-specific malaria cases per 100,000 inhabitants per year. For *P. falciparum*, all monoinfections and mixed infections were considered as *P. falciparum* infections, but only *P. vivax* monoinfections were included for calculating *P. vivax* rates. Data for mixed infections have been reported separately from *P. falciparum* infections since 1994, but in previous years, these two were consolidated as a single figure and so reported. Years of countries for which data for a variable were missing were removed from the analysis. API and species-specific rates were calculated using the total population of the country.

The population living at risk of malaria transmission should be used for calculation of API; however, there are vast differences in the manner in which countries estimate and report their at-risk population. The population in a locality or focus where malaria transmission occurs should be used to determine those at risk; nevertheless, it is logistically impractical in a highly endemic area. Population of the whole municipality where malaria is reported is considered at risk for reporting purposes, but not all municipalities have the same risk; therefore, the whole population may or may not be at risk. To avoid this bias, total country population instead of reported population at risk was used. API and SPR are not independent and may be expected to show similar trends given adequate quality of surveillance; thus, analysis of both indices allows detection of true trends and surveillance quality.

Joinpoint methodology has been used for trend analysis in cancer rates by the National Cancer Institute of the United States.^16^ The regression method tests for the possibility that two or more linear regression curves can be fitted for different ranges of the independent variables (i.e., in this case, one long period for a country can be possibly divided into two or more periods, each with a different trend). The slope of the linear curve or annual percentage change (APC) in disease incidence is different for each of the smaller periods; each time point (year) that divides the longer period into shorter periods where a significant change in APC is detected is considered to be a joinpoint.

SEs of the rates were calculated assuming Poisson distribution for incidence rates and binomial distribution for percentages. The data were analyzed using the Joinpoint program.^17^ A regression analysis using logarithmic rates as the dependent variable and years as the independent variable was carried out. A factor of 0.1 was added to all of the years in a data series of a variable for a country where a zero value was observed in 1 or more years. Heteroscedasticity was assumed, and the SEs calculated were used for the regression. An autocorrelated errors model was fitted for calculation of regression estimates. A minimum of 4 years between two subsequent joinpoints and 3 years before the first joinpoint were set. Permutation tests up to 4,999 were used to identify the best model. Results were considered to be significant at *P* value less than 0.05. Numbers of joinpoints, final selected model, APC for all years, and average APC (AAPC) for the period 2000–2011 were calculated and have been reported.

## Results

Of 21 countries for which data were analyzed, annual malaria case series were available for all four variables over 53 years from 1959 to 2011 for 14 countries (Table 1). For Argentina, SPR data were not available for 2009. Information from Brazil was available for all variables during the entire period with the exception of the year 1959. Information on SPR for Colombia was unavailable for 1998, and information on SPR for French Guiana was unavailable for 1959 and 1998. Similarly, the rate was unavailable for Guatemala in 1998 and Peru in 1989 and 2011. Species-specific rates were unavailable for French Guiana in 1959. For Haiti, data were available since 1962, and information for all variables was missing for 1995, 2002, and 2003. Overall, these instances of data unavailability added up to 37 years per country per indicator, accounting for missing data of less than 1% (0.83%) of the possible total of 4,452 years per country per indicator.

### Trends in malaria incidence.

**Figure 1.**
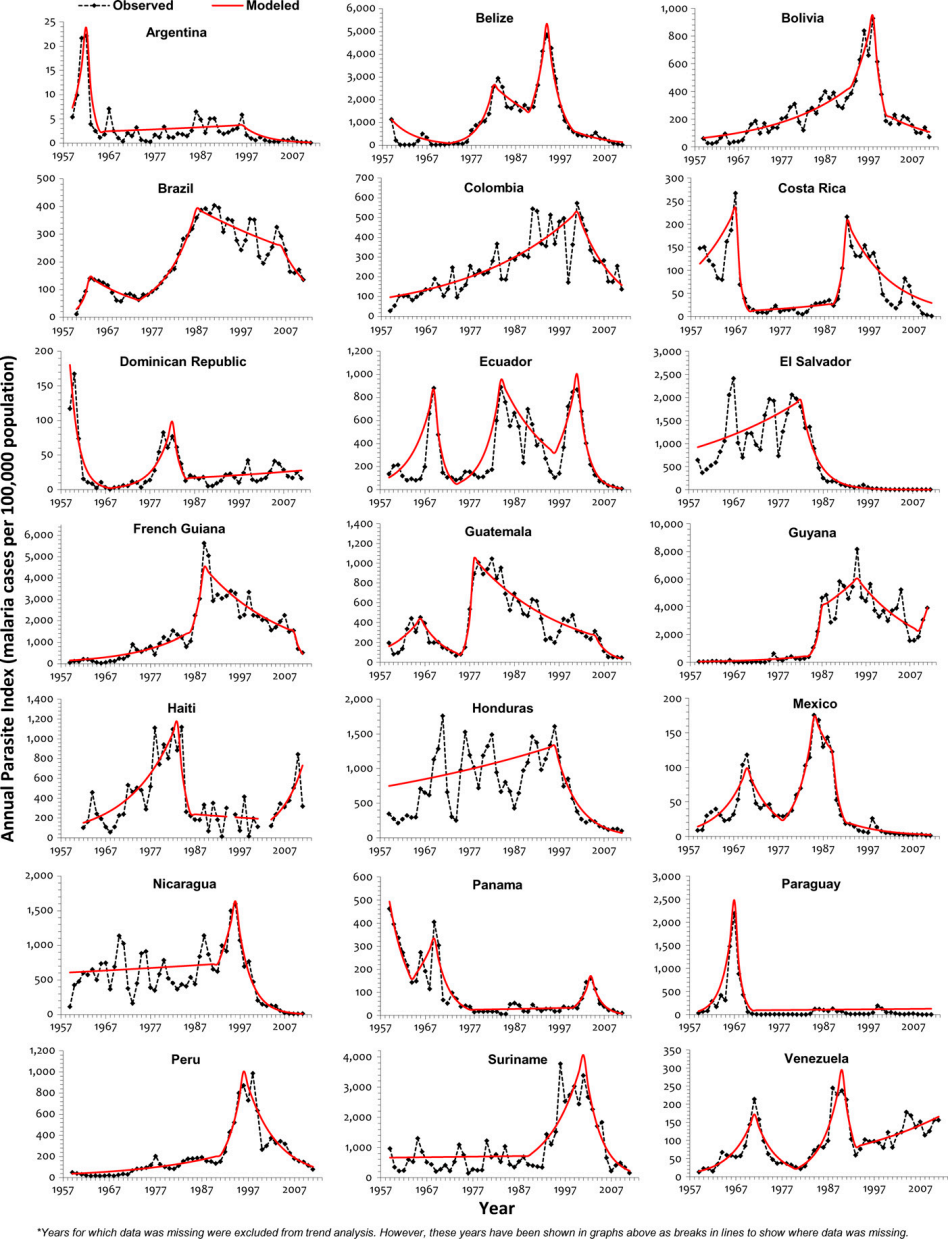
Malaria trend in endemic countries of the Americas, 1959–2011.

For the majority of countries, the trends of the principal indicators API and SPR were similar over most of the five decades studied, although they did vary in magnitude (Figures 1

and 2

**Figure 2.**
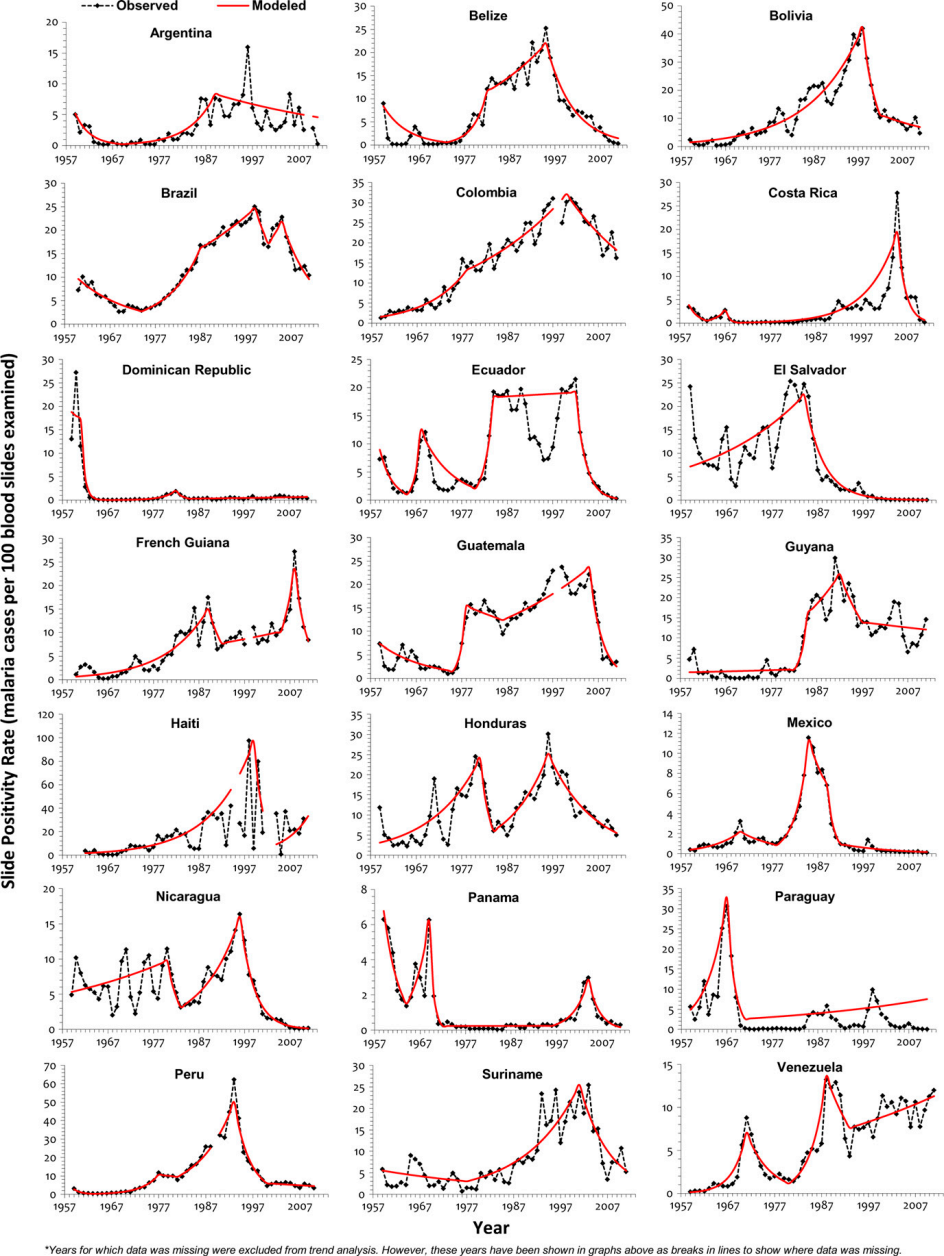
Trend of SPR for malaria in the Americas, 1959–2011.

). In French Guiana and Guatemala, the trends for these two indicators were widely different and even in different directions. Specifically, in Guatemala, whereas API was declining significantly, SPR was increasing significantly between 1986 and 2005, pointing to a possible decrease in surveillance quality and coverage of diagnosis and treatment during those years. Mexico, El Salvador, and Paraguay were the only countries where the joinpoint years and trend directions for both SPR and API coincided for almost all segments, but different values were obtained for others (Table 2). For Guyana, no significant (*P* value < 0.05) SPR trend was seen for the entire period.

During 1960–1969, most countries had significantly increasing API (*P* value < 0.05), and for a majority of these years, most countries also had a significantly increasing SPR. Malaria incidence (SPR and API) continued to increase throughout the 1970–1979 period and peaked in the early 1980s, when only Guatemala had a significant reduction in API and none of the countries had a significant declining SPR (Supplemental Tables 1–4). By 1989, a reversal in the malaria trends was observed; five countries had significantly decreasing API, but it was not accompanied by significant reductions in the SPR. During 1990–1999, this declining malaria trend was further accentuated; by the end of that decade, 13 countries showed reductions in API, and the number of countries with significantly declining SPR increased from 2 to 7. Malaria continued to decline, and by the middle of 2000–2011, 12 and 16 countries had significantly decreasing SPR and API, respectively. Between 2011 and the previous joinpoint year, only Haiti and Venezuela had significant increases in API, whereas Venezuela and the Dominican Republic also had significant increases in SPR. The change from an increasing or non-significant trend for API to a significantly declining API or joinpoint year was seen in the 1990–1999 period for most countries in the Americas, but in Colombia, Ecuador, Panama, and Suriname, this change was observed in the 2000–2011 period. In some countries, the significant declining API has not been of constant magnitude; in Belize, the annual rate of reduction declined from 29.69% in the 1994–2000 period to 12.89% in the subsequent period. In Bolivia, there was a similar change in the rate of decline from 37.22% between 1998 and 2001 to 7.44% since 2001. The rapid API reduction rates were consequent of the fact that, for the 53-year period under review, both of these countries reported the highest API during 1990–1999, and the initial rapid decline was from those observed peaks. In contrast, the annual rate of decline increased in Brazil and Guatemala from 2.20% and 4.95% to 12.20% and 28.78%, respectively, with the joinpoint year being 2005 for Guatemala and 2006 for Brazil. A major reversal of malaria trend toward decline was seen in the 2000–2011 period; 13 countries had a significantly declining SPR in 2011, and 7 of them (Brazil, Costa Rica, Ecuador, French Guiana, Guatemala, Panama, and Suriname) changed from previously increasing or non-significant SPR during 2000–2011. Of note is the significantly declining SPR for over two decades in Mexico and El Salvador, both of which are presently in the pre-elimination phase.

### Species-specific trends.

**Figure 3.**
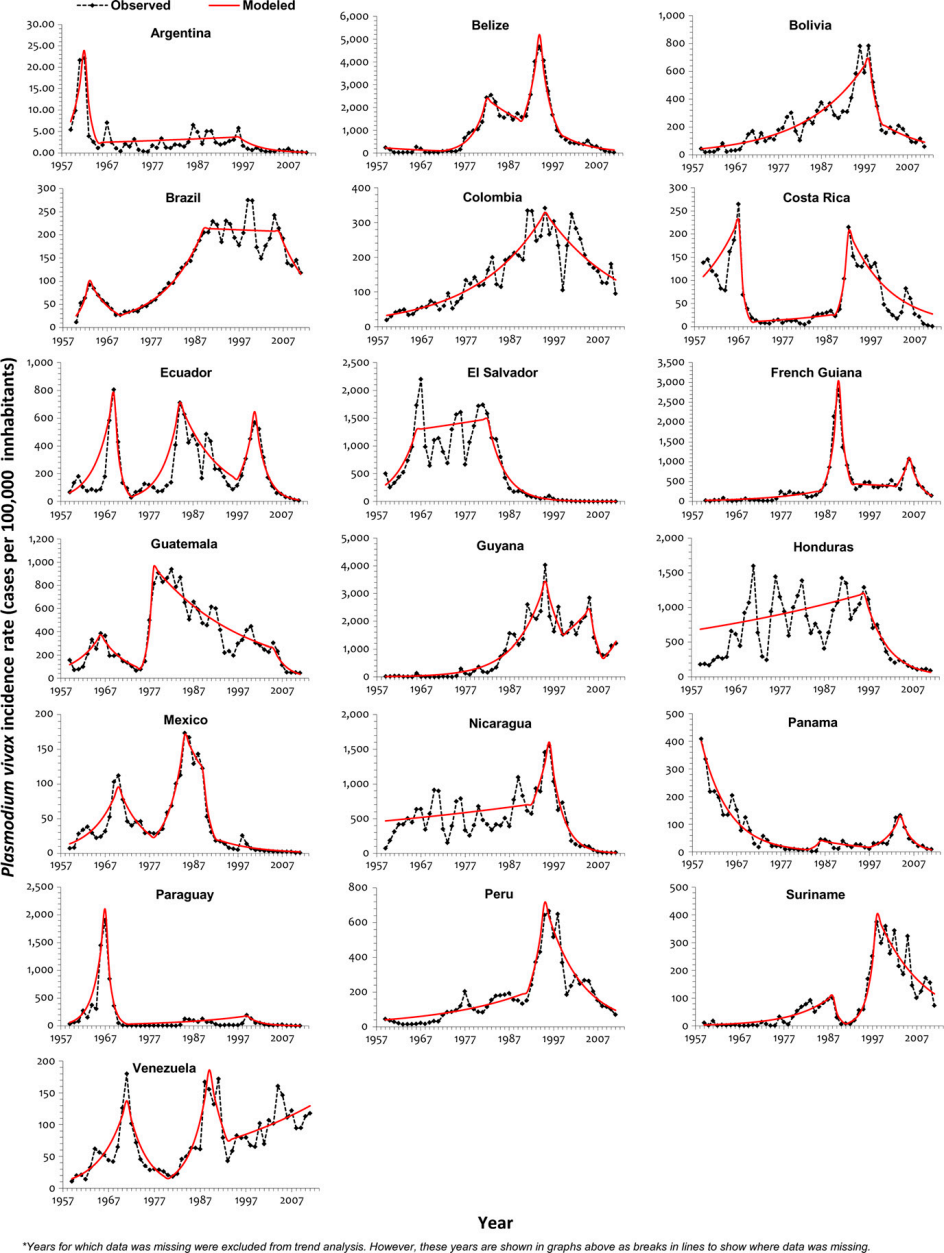
Trend of PvIR (per 100,000 inhabitants) in the Americas, 1959–2011.

Analysis of the *P. falciparum* incidence rate (PfIR) was not undertaken for Argentina and analysis of the *P. vivax* incidence rate (PvIR) was not undertaken for Haiti and Dominican Republic, because the number of malaria cases for the respective species was zero in those countries for most of the years. Trends in PvIR and PfIR were almost similar in Bolivia, Paraguay, and Venezuela (Figures 3

and 4

**Figure 4.**
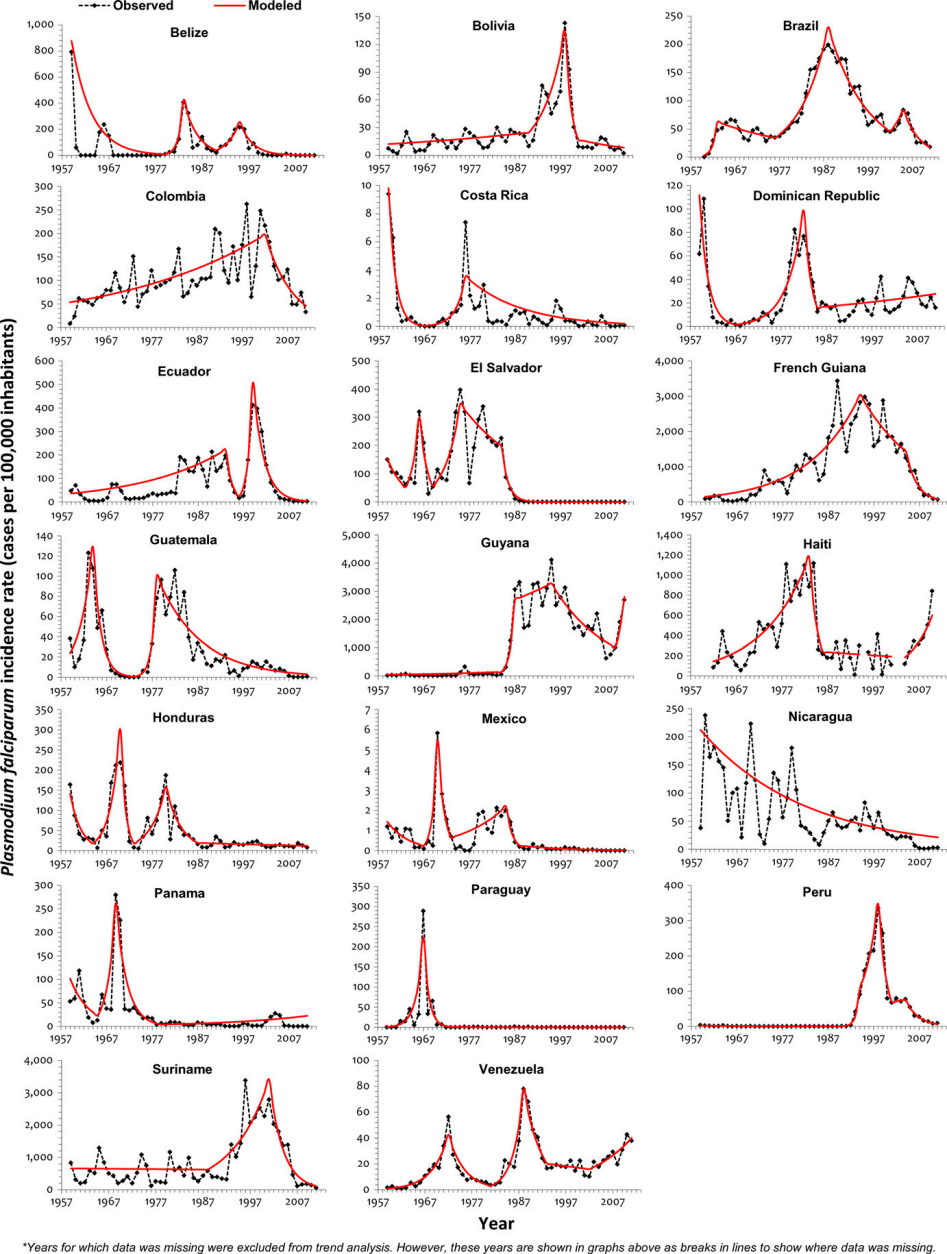
Trend of PfIR (per 100,000 inhabitants) in the Americas, 1959–2011.

). Joinpoint years for the two species-specific rates were not equal for any of the countries, signifying different species-specific trends for all countries (Table 3). There was no joinpoint in the PfIR for Nicaragua, but a significant reduction (*P* value < 0.05) was observed throughout the 53-year period studied. Similar to API and SPR trends, PvIR increased significantly in a number of countries during 1960–1985. Thereafter, a reversal was seen in subsequent years. PvIR reduced significantly in many countries in the latter half of 1990–1999 and almost all countries^17^ by the mid-2000–2011 period. By 1999, one-half of the countries had significantly declining PfIR, and this continued through 2000–2011. Increasing PfIR and PvIR have been observed in Guyana from 2008–2011. Argentina, El Salvador, Mexico, and Paraguay have not reported autochthonous *P. falciparum* cases in the past decade.

### Trends (AAPC) since 2000.

**Figure 5.**
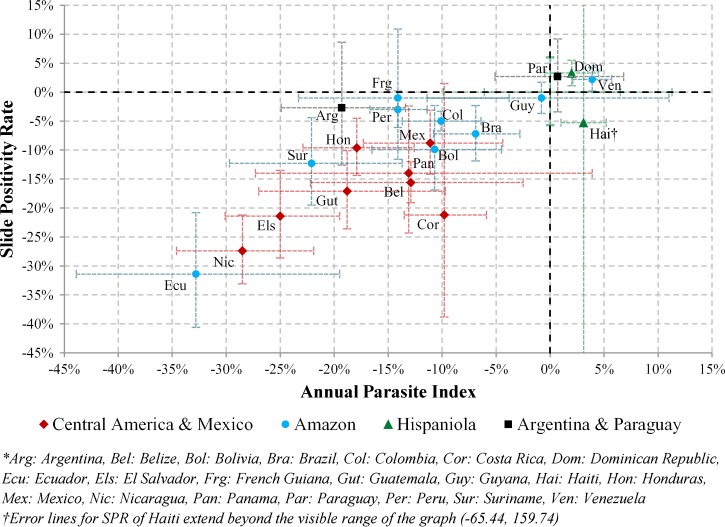
AAPC in API and SPR by subregion for 2000–2011.

Significant decreases (*P* value < 0.05) in the AAPC of the API were observed in 16 of 21 endemic countries during the 2000–2011 period (Figure 5

). In 11 of 16 countries, significant decreases in AAPC for the SPR were also observed. In the Mesoamerican subregion, there was a significant decline in malaria (Belize, Costa Rica, El Salvador, Guatemala, Honduras, Nicaragua, and Mexico), with reductions in the API of over 9% annually, and with the exception of Costa Rica, there were significant decreases in SPR, being higher than 8% annually (Table 4). This rapid and sustained decline in the whole subregion in the last decade has formed the basis for present regional malaria elimination efforts in these countries. Five of eight countries that share the Amazon Basin in South America, namely Bolivia, Brazil, Colombia, Ecuador, and Suriname, had significant reductions in AAPC for both API and SPR. In Ecuador, malaria declined at a very rapid pace: more than 30% annual decreases in both API and SPR. Suriname, El Salvador, and Nicaragua were other countries where malaria declined significantly: over 20% annual decline in API. Analyses for Paraguay revealed no significant trends for either the SPR or API, but after removing the epidemic years of the 1960s, significant decline in both the API and SPR was observed in recent years (2000–2011). However, API increased by over 3% annually in Haiti and Venezuela. Even in absolute numbers, the case burden has decreased since the last joinpoint for most countries in the American continents (Table 5).

## Discussion

Trends presented here are based on reported cases, which are affected by robustness of surveillance systems and quality and coverage of diagnostic services. However, trends for most countries and periods in our study seem to generally reflect the historic incidence of disease. Several factors may be responsible for malaria increases in these years: some specific to countries and others specific to certain periods of time. In general, malaria increased during the first decade of the eradication efforts in the 1950s in the Americas, probably associated with improved case detection and reporting. Malaria spiked in the 1970s as vector resistance to insecticides, primarily dichloro diphenyl trichloroethane (DDT), became established in most Central American countries; between 1975 and 1978, malaria incidence increased by almost 11 times in Guatemala because of insecticide resistance.^18^ However, drug resistance was responsible for sporadic outbreaks in those years in South America; introduction of chloroquine-resistant *P. falciparum* from the Marowijne region of Suriname to the Upper Suriname river area led to a fivefold increase in cases in that area in 1974 and 1975.^19^ Hurricanes David and Federico in 1979 were reasons for increases in parts of Central America and the Caribbean countries.^20^

Disruptions in health services during political and social disturbances were responsible for increases in some countries; decrease and cessation of vector control activities by 1979 in Nicaragua because of the revolution led to an accompanying increase in malaria.^21^ Areas of this country affected by Contra attacks reported an increase in cases, whereas other unaffected areas reported decreases during 1983–1985, leading to only a slight change in malaria incidence overall. Change of agricultural practices was a principal reason for the increase in malaria in the 1990s in many Central American countries; switching from growing rice and cotton to African palm and banana was associated with an increase in breeding sites caused by deforestation and associated work-related migration of people, often from malaria-endemic areas.^22^ A 10-fold increase was reported in Costa Rica between 1989 and 1992 because of an earthquake in 1991, and as the banana plantation industry flourished in the Huetar Atlantic Region of the country, malaria transmission shifted from the Pacific to the Atlantic coast during 1991–1999.^20^,^23^ Most countries in South America, excluding Ecuador, had increased malaria incidence during 1993–1995, because indoor residual sprayings with DDT were decreased or abolished.^24^ Decentralization of health systems, integration of the vertical national malaria eradications programs into the system, displacement of health personnel dedicated to combat malaria, and reduced malaria-specific resources are among the factors associated with increases in malaria in most countries during different periods.^25^

Reduction in malaria started well in the 1990–1999 period and continued throughout 2000–2011. However, some countries had the highest malaria incidence rates of the five decades during the 1990–1999 period, including Belize, Bolivia, Costa Rica, Guyana, Nicaragua, Peru, and Suriname. Other countries also reported spikes in malaria incidence in the same period. Some of these were specific to the El Niño Southern Oscillation effect, especially the peak observed in 1996 in Colombia, Ecuador, and Peru. It has been previously shown that this environmental phenomenon has been associated with malaria epidemics in Colombia, Peru, Venezuela, and Guyana in the 1970s and 1980s.^26^–^28^ Malaria funding also declined regionally in the 1990s; between 1992 and 1998, funds available per person for malaria control declined from US$1.10 to US$0.40.^29^

The majority of the countries had simultaneous significant downtrends for both API and SPR during the 2000–2011 period, which can be considered a demonstration of a declining malaria incidence accompanied by consistently functional surveillance. This is related to increased malaria control and surveillance efforts in most endemic countries, 15 of which received external financial support through Global Fund for AIDS, Tuberculosis and Malaria (Global Fund) grants during that period. Policy changes and improvement in implementation of interventions based on evidence generated within the ambit of the Amazon Malaria Initiative (AMI) supported by the US Agency for International Development and with technical cooperation from PAHO, Centers for Disease Prevention and Control, Management Sciences for Health, US Pharmacopeia, Research Triangle Initiative, and Links Media have also led to this decrease.^30^

Venezuela, Dominican Republic, and Haiti show an increasing malaria incidence in recent years. In Venezuela, this is accompanied by an increasing proportion of cases caused by *P. falciparum.* Adjoining areas of Guyana have been presenting a similar picture in recent years associated with increased gold mining in the Amazon forest areas of these two countries. This phenomenon has also increased the population at risk of malaria and in most instances, in virgin rainforests with limited access to health clinics. This underlines the shared risk factors of mobile populations living at the high risk associated with gold mining in these two countries as well as Suriname, French Guiana, and adjoining areas of Brazil.^31^–^33^ Malaria has declined in all other countries sharing the Amazon Basin. Brazil and Colombia together report more than one-half of the malaria cases in the Americas and have shown efforts in identifying and successfully targeting at-risk populations.^13^,^34^–^36^ A project for malaria in the Andean region across international boundaries was successful in reduction of the disease and reinforced the need for such efforts.^37^ *P. falciparum* malaria declined in the countries sharing the Amazon Basin that all changed the first line of treatment to artemisinin-based combination therapies during 2000–2011 based on evidence generated by therapeutic efficacy studies within the Amazon Network for the Surveillance of Antimalarial Drug Resistance (RAVREDA) initiative supported by AMI. However, *P. vivax* malaria also declined at the same time, pointing to the probable impact of other interventions for malaria control or changes in socioeconomic and/or environmental factors that were at play. However, additional studies are required for analyzing these associations.

Haiti and Dominican Republic, sharing the island of Hispaniola, have exclusively *P. falciparum* transmission and present a challenge, because they are the only focus of continued malaria transmission in the Caribbean. A large proportion of cases in Dominican Republic has reportedly been imported from Haiti, leading to an increase in the total case incidence, although the number of autochthonous cases in the country has been declining. Natural disasters have been associated with increases in malaria incidence on the island; Hurricanes David and Federico led to increase in 1981–1982 and Hurricane George led to an increase in 1998 in Dominican Republic. Recently, the Haiti earthquake in 2010 led to a 67% increase compared with 2009. It is likely that this is an artefact, because the increase was mostly from the center department of the country, which was most affected by the earthquake, and the same persons may have been tested more than one time by different relief agencies, leading to duplication of data. Limited access to diagnosis and treatment and a weak surveillance system increase the uncertainty of observed malaria incidence trends in the country; for example, the SPR reported by the country varied from 97.4% in 1998 to 5.6% in 1999.^38^ Malaria cases imported from this country have been associated with outbreaks in other Caribbean countries; conditions for malaria transmission exist in many other island nations.^39^

Mexico, El Salvador, and Costa Rica, three countries in the pre-elimination phase, have had sustained reductions in malaria incidence for two or more recent decades. They do not report autochthonous *P. falciparum* cases, and malaria is limited to focal areas of these countries.^40^,^41^ In other Central American countries, namely Belize, Guatemala, Honduras, Nicaragua, and Panama, malaria has also declined significantly, and the disease is now focalized in pockets of the subregion. Although Honduras and Nicaragua have been reporting higher proportions of cases caused by *P. falciparum* in the last decade, this is considered a result of increased surveillance in areas with previously greater inaccessibility in the shared La Moskitia region between the two countries. All of the Central American countries are preparing to progress toward the pre-elimination and elimination phases. Political support for this goal was evidenced in the form of a recent resolution passed by the ministers of health of the Mesoamerican countries as well as the Dominican Republic and Haiti to eliminate malaria by 2020.^42^

Argentina and Paraguay, in the southern cone of South America, are also targeted for elimination; they are both presently in the pre-elimination phase with exclusively *P. vivax* transmission. Paraguay reported an outbreak of malaria in 1999, but thereafter, malaria decreased significantly, with only one autochthonous case reported in 2011 and none up to week 51 in 2012.^43^ Its efforts at decreasing malaria were recognized, and the national malaria control program of the country was awarded the Malaria Champion of the Americas in the year 2012.^44^

Overall, funding for malaria control from national sources kept increasing while financing from external sources declined between 1957 and 1986 with waning interest of donor agencies, largely mirroring the malaria problem as it grew during those years.^22^ In particular, in 1988 in Haiti, a major financial crisis led to closing of the National Malaria Eradication Program.^38^ However, funding for malaria control has been increasing again since 2000 from both national sources and external aid, particularly since 2002 through the Global Fund, and it has been accompanied with a decline in malaria in most countries. However, additional investigation is required to adeptly relate the malaria trend in the American continents to historic trends in malaria financing and coverage of other malaria control interventions. The new funding model of the Global Fund takes the countries' World Bank classification based on the gross domestic product (GDP) and malaria burden into account. Thus, many previously eligible countries in the Americas will have less likelihood of receiving external funding. Given the fact that 18 of the endemic countries have shown marked decreases in malaria burden and some of these have also intimated interest in eliminating the disease, it is imperative that national resources presently available continue to be destined to further malaria reduction. Also, additional resources should be made available to ensure functional surveillance systems in areas where elimination has been achieved to prevent reintroduction. Failure to adequately address funding shortages in years ahead could lead to increases in malaria, which has been previously seen.

In a 2011 article, White^45^ proposed that increased *P. falciparum* incidence could lead to an increase in *P. vivax* because of the activation of hypnozoites in areas where transmission of the two species occurs simultaneously. Such a temporal increase pattern was seen in Brazil and Venezuela during the 1985–1988 period and Ecuador during 1997–1999. However, such an association can be better ascertained with data collected at weekly or monthly intervals and at the level of localities or foci. Associations like these will require more detailed analysis to better establish this relationship, because the data presented here do not so permit.

Data for some years were missing in our study (< 1%), but the proportion of unavailable data points was small, and as such, the effect of missing data is considered to be minimal. Although Chaparro and Padillo^46^ used joinpoint regression analysis for malaria deaths in Colombia, this methodology has mostly been used for chronic diseases, such as cancer, which unlike malaria, are not prone to rapid changes in annual incidence rates.^46^–^52^ Thus, epidemic years could significantly affect the malaria trend, which was the case in Paraguay, where an otherwise non-significant trend in recent years changed to a significant decline on removing the epidemic year of 1969 and previous years. Furthermore, the innate variability in reporting of incidence data because of social, economic, and programmatic reasons also limits interpretation of trends. However, use of joinpoint regression in this study has provided satisfactory results. Decreased cases were reported in 1996 and 1997 in Guatemala owing to temporary weakening of information systems as a result of decentralization; however, this artefact did not affect the declining trend since the late 1970s that was evidenced by joinpoint methodology. However, large and abrupt variation in cases, which was seen in some countries in the 1960s and 1970s, resulted in non-significant trends, signifying possible inadequate quality of data. This suggests that joinpoint methodology is robust and may also be considered for other infectious diseases, like tuberculosis and human immunodeficiency virus/acquired immune deficiency syndrome (HIV/AIDS).

Although this study used country-level data, malaria incidence rates could both increase and decrease at the state or district level within a country in the same year or period. State- or district-level analyses would, thus, present a better understanding of underlying patterns of malaria incidence for use in decision-making at the subnational and local levels, especially when pursuing the goal of malaria elimination. API depends on the definition of the population at risk; the population of the whole country is not accurate for calculating API, because it underestimates the incidence. SPR is conservative, because it measures incidence of malaria in only suspected or tested cases, which are always fewer than the total population at risk. Although both the definition of a suspected case and the coverage of diagnosis impact SPR, it has been proven to be a reliable indicator for measurement of malaria incidence trends.^53^ Simultaneous analyses of both API and SPR provide more reliability in trends, because convergent trends in these two indicators signify actual decline in malaria incidence, whereas divergent trends could be caused by inadequate information or decreases in coverage of diagnosis. This is well-observed between 1986 and 2004 in Guatemala, where declining API would give an impression of decreasing malaria, although increasing SPR points to the problems of decreases in diagnosis and treatment coverage and associated surveillance quality. National surveillance systems and coverage of the national malaria programs have improved over the past 50 years in the endemic countries of the Americas.^54^,^55^ Countries of the Americas have moved from reporting of malaria cases as aggregate data to nominal case reporting, thereby decreasing the chances of duplication of data and improving the quality of data. Presently, all countries, except Haiti, have nominal reporting systems implemented completely or partially throughout the country. Laboratory-confirmed cases have been used in this study; improvement in the coverage and quality of diagnostic services over the years is, thus, a limiting factor in interpreting results. Furthermore, improvements in reporting completeness and timeliness and changes in surveillance systems, definitions, and health-seeking behavior also limit comparison of results over various decades. Recent annual incidence rates are more specific than those of the previous decades, which could lead to an under- or overestimation of trends. Use of annual incidence estimates adjusted for these improvements over the study period would have prevented this error; however, this is beyond the scope of this study. Factors affecting these historical trends and especially, those during the 2000–2011 period will be further analyzed and presented separately in a future manuscript.

The majority of the endemic countries on the American continents had significantly decreasing trends in malaria incidence in recent years. Although such significant decreases have been seen in previous years in several of these countries, in the past, a sustained decline spanning more than one decade and more so, in almost all of the countries at the same time has been uncommon. The recent achievements are considered a reflection of sustained efforts of country programs and increased funding for malaria and vector-borne disease control accompanied by political will and commitment at the national and global levels.^2^,^14^,^56^,^57^ The sustained focus on malaria through the establishment and monitoring of MDGs has been an important catalyst for this decline, and it gives credence to the suggestion that the post-MDG agenda should include goals for further decline and possible elimination of malaria. The current PAHO strategy and plan of action for malaria in 2011–2015 took the declining trend into account and included malaria elimination where feasible as a goal. It will transition into the post-2015 strategy and plan of action, which will be aligned with the global technical strategy for malaria being developed by the WHO. In that context, for the American continents, the current situation presents a unique opportunity to develop multicountry malaria elimination efforts as models for other parts of the world, which also highlight and underline the importance of sustained efforts for malaria control and its possible elimination in the Americas. However, newer challenges have emerged, including the recent report of a suspected decrease in artemisinin efficacy in Guyana and Suriname, which has necessitated immediate steps for the control of malaria in the Guiana Shield.^58^,^59^ Although the reported trends in the last decade can catalyze further efforts to eliminate the disease from the Americas, as the malaria burden decreases, the lessons of reintroduction in Jamaica in 2006 and the need for constant surveillance after elimination must not be forgotten.^39^

## Supplementary Material

Supplemental Tables.

## Figures and Tables

**Table 1:** Data availability by parameter evaluated and country, 1959–2011

Country	Range of years	Number of data years available			
		API	SPR	PfIR	PvIR
Argentina	1959–2011	53	52	53	53
Belize	1959–2011	53	53	53	53
Bolivia	1959–2011	53	53	53	53
Brazil	1960–2011	52	52	52	52
Colombia	1959–2011	53	52	53	53
Costa Rica	1959–2011	53	53	53	53
Dominican Republic	1959–2011	53	53	53	53
Ecuador	1959–2011	53	53	53	53
El Salvador	1959–2011	53	53	53	53
French Guiana	1959–2011	53	51	52	52
Guatemala	1959–2011	53	52	53	53
Guyana	1959–2011	53	53	53	53
Haiti	1962–2011	47	47	47	47
Honduras	1959–2011	53	53	53	53
Mexico	1959–2011	53	53	53	53
Nicaragua	1959–2011	53	53	53	53
Panama	1959–2011	53	53	53	53
Paraguay	1959–2011	53	53	53	53
Peru	1959–2011	53	51	53	53
Suriname	1959–2011	53	53	53	53
Venezuela	1959–2011	53	53	53	53

**Table 2:** APC in API (per 100,000 inhabitants) and SPR (per 100 blood slides examined) in the Americas, 1959–2011

Country	API			SPR		
	Number of joinpoints	Year range	APC (%; 95% confidence interval)	Number of joinpoints	Year range	APC (%; 95% confidence interval)
Argentina	3	1959–1962	48.14 (−3.74, 127.98)	2	1959–1969	−27.23 (−34.13, −19.60)
		1962–1965	−53.25 (−88.38, 88.08)		1969–1989	20.07 (13.82, 26.67)
		1965–1996	1.38 (−1.03, 3.85)		1989–2011	−2.67 (−8.23, 3.22)
		1996–2011	−19.26 (−29.14, −8.00)			
Belize	5	1959–1972	−17.49 (−27.31, −6.34)	3	1959–1973	−17.11 (−26.32, −6.75)
		1972–1982	39.69 (19.76, 62.95)		1973–1982	38.81 (16.58, 65.29)
		1982–1990	−7.01 (−13.35, −0.21)		1982–1995	5.02 (2.29, 7.83)
		1990–1994	37.96 (14.65, 66.00)		1995–2011	−15.56 (−19.02, −11.95)
		1994–2000	−29.69 (−36.96, −21.58)			
		2000–2011	−12.89 (−22.18, −2.49)			
Bolivia	3	1959–1993	5.79 (4.22, 7.39)	2	1959–1998	8.92 (7.59, 10.27)
		1993–1998	16.70 (2.32, 33.10)		1998–2002	−28.00 (−44.01, −7.41)
		1998–2001	−37.21 (−58.28, −5.49)		2002–2011	−5.31 (−13.04, 3.12)
		2001–2011	−7.46 (−13.15, −1.39)			
Brazil	4	1960–1963	71.28 (−21.41, 273.27)	5	1960–1974	−8.59 (−11.27, −5.82)
		1963–1974	−7.45 (−13.48, −1.01)		1974–1987	14.64 (12.27, 17.06)
		1974–1987	15.23 (11.10, 19.50)		1987–1999	3.58 (2.22, 4.96)
		1987–2006	−2.20 (−3.36, −1.03)		1999–2002	−11.22 (−23.47, 2.98)
		2006–2011	−12.16 (−20.19, −3.32)		2002–2005	8.17 (−7.21, 26.10)
					2005–2011	−12.82 (−16.02, −9.50)
Colombia	1	1959–2001	4.12 (3.26, 4.99)	2	1959–1978	11.86 (9.61, 14.16)
		2001–2011	−11.39 (−15.32, −7.28)		1978–2000	4.11 (3.24, 4.99)
					2000–2011	−5.02 (−6.68, −3.33)
Costa Rica	4	1959–1967	9.39 (−2.28, 22.46)	4	1959–1963	−38.97 (−55.81, −15.70)
		1967–1970	−62.83 (−86.39, 1.52)		1963–1967	49.47 (−1.13, 125.95)
		1970–1989	4.47 (−4.2, 13.93)		1967–1970	−67.22 (−88.08, −9.89)
		1989–1992	95.53 (−6.44, 308.61)		1970–2005	16.42 (12.30, 20.70)
		1992–2011	−9.82 (−13.53, −5.94)		2005–2011	−43.06 (−64.70, −8.14)
Dominican Republic	3	1959–1968	−38.86 (−49.09, −26.56)	4	1959–1961	−4.47 (−44.90, 65.62)
		1968–1982	31.35 (18.29, 45.86)		1961–1967	−70.99 (−81.70, −54.01)
		1982–1985	−44.67 (−76.95, 32.79)		1967–1982	41.92 (29.79, 55.18)
		1985–2011	2.00 (−0.49, 4.54)		1982–1985	−46.61 (−74.81, 13.14)
					1985–2011	3.25 (1.09, 5.46)
Ecuador	5	1959–1969	23.48 (4.78, 45.52)	5	1959–1965	−30.1 (−48.53, −5.07)
		1969–1974	−43.68 (−65.18, −8.91)		1965–1968	128.05 (−13.03, 497.99)
		1974–1984	34.53 (15.14, 57.19)		1968–1980	−13.78 (−20.23, −6.81)
		1984–1996	−8.71 (−15.48, −1.40)		1980–1984	72.02 (24.22, 138.22)
		1996–2001	25.73 (−3.5, 63.82)		1984–2002	0.25 (−1.65, 2.19)
		2001–2011	−36.89 (−48.55, −22.60)		2002–2011	−36.95 (−47.39, −24.45)
El Salvador	1	1959–1982	3.29 (1.05, 5.58)	1	1959–1984	4.68 (2.71, 6.68)
		1982–2011	−24.98 (−30.08, −19.51)		1984–2011	−21.42 (−28.58, −13.54)
French Guiana	3	1959–1986	8.85 (4.35, 13.55)	4	1960–1989	11.30 (8.10, 14.70)
		1986–1989	44.01 (−9.05, 128.01)		1989–1992	−19.90 (−42.70, 12.10)
		1989–2009	−5.35 (−6.61, −4.08)		1992–2005	2.20 (−0.50, 5.00)
		2009–2011	−44.61 (−70.69, 4.67)		2005–2008	32.20 (−4.60, 83.20)
					2008–2011	−29.70 (−46.70, −7.40)
Guatemala	4	1959–1966	15.26 (−4.35, 38.89)	4	1959–1975	−9.32 (−13.87, −4.53)
		1966–1975	−17.33 (−29.42, −3.18)		1975–1978	115.70 (2.02, 356.05)
		1975–1978	136.99 (4.20, 438.99)		1978–1986	−2.65 (−7.65, 2.61)
		1978–2005	−4.95 (−6.19, −3.70)		1986–2005	3.45 (1.78, 5.14)
		2005–2011	−28.78 (−41.77, −12.89)		2005–2011	−31.06 (−40.88, −19.61)
Guyana	4	1959–1984	9.32 (1.34, 17.92)	4	1959–1982	1.68 (−6.92, 11.07)
		1984–1987	104.49 (−22.2, 437.45)		1982–1985	93.97 (−78.52, 1651.59)
		1987–1995	5.24 (−1.82, 12.80)		1985–1992	6.99 (−2.13, 16.96)
		1995–2009	−6.78 (−9.69, −3.77)		1992–1997	−11.66 (−24.74, 3.69)
		2009–2011	31.50 (−29.43, 145.01)		1997–2011	−1.01 (−3.68, 1.72)
Haiti	3	1962–1983	10.20 (7.40, 13.10)	2	1962–1999	11.61 (9.98, 13.26)
		1983–1986	−40.88 (−82.8, 102.9)		1999–2004	−37.55 (−96.36, 971.81)
		1986–2004	−1.47 (−6.50, 3.80)		2004–2011	20.22 (−0.65, 45.47)
		2004–2011	21.75 (9.60, 35.30)			
Honduras	1	1959–1996	1.56 (0.02, 3.13)	3	1959–1981	9.76 (5.89, 13.78)
		1996–2011	−17.86 (−22.83, −12.56)		1981–1984	−36.63 (−64.07, 11.79)
					1984–1996	12.55 (6.49, 18.94)
					1996–2011	−9.63 (−14.46, −4.51)
Mexico	5	1959–1970	19.26 (9.51, 29.88)	5	1959–1970	16.98 (8.81, 25.76)
		1970–1978	−16.55 (−25.62, −6.37)		1970–1978	−10.99 (−19.62, −1.44)
		1978–1985	33.24 (18.61, 49.67)		1978–1985	44.37 (30.41, 59.83)
		1985–1989	−8.47 (−22.61, 8.25)		1985–1989	−11.77 (−24.29, 2.82)
		1989–1992	−44.16 (−68.6, −0.67)		1989–1992	−47.91 (−70.46, −8.15)
		1992–2011	−11.11 (−17.34, −4.41)		1992–2011	−8.81 (−14.16, −3.13)
Nicaragua	2	1959–1992	0.56 (−1.01, 2.16)	3	1959–1980	2.92 (−0.50, 6.45)
		1992–1996	22.26 (−5.75, 58.59)		1980–1983	−31.22 (−67.46, 45.4)
		1996–2011	−28.46 (−34.53, −21.82)		1983–1996	13.24 (8.25, 18.46)
					1996–2011	−27.44 (−33.18, −21.22)
Panama	5	1959–1964	−20.44 (−30.74, −8.61)	5	1959–1964	−25.91 (−36.26, −13.9)
		1964–1969	16.18 (−11.35, 52.27)		1964–1969	32.49 (−0.14, 75.77)
		1969–1977	−27.92 (−36.63, −18)		1969–1972	−66.83 (−85.3, −25.16)
		1977–2000	1.34 (−2.80, 5.66)		1972–1996	0.25 (−3.36, 4.00)
		2000–2004	50.90 (−1.07, 130.17)		1996–2004	36.32 (17.16, 58.61)
		2004–2011	−36.56 (−46.03, −25.44)		2004–2011	−33.85 (−45.14, −20.24)
Paraguay	2	1959–1967	55.07 (25.65, 91.38)	2	1959–1967	26.82 (4.89, 53.34)
		1967–1971	−55.73 (−74.52, −23.08)		1967–1971	−46.79 (−68.81, −9.22)
		1971–2011	0.73 (−5.36, 7.22)		1971–2011	2.67 (−3.11, 8.79)
Peru	2	1959–1991	5.59 (2.39, 8.88)	5	1959–1963	−46.2 (−68.22, −8.90)
		1991–1996	36.45 (16.80, 59.41)		1963–1977	31.7 (22.26, 41.88)
		1996–2011	−14.10 (−16.73, −11.38)		1977–1981	−2.58 (−31.25, 38.04)
					1981–1993	14.81 (10.71, 19.06)
					1993–2000	−26.09 (−29.03, −23.03)
					2000–2010	−2.97 (−6.03, 0.19)
Suriname	2	1959–1989	0.27 (−2.68, 3.31)	2	1959–1977	−3.29 (−8.80, 2.57)
		1989–2001	15.51 (6.04, 25.83)		1977–2001	9.29 (6.53, 12.11)
		2001–2011	−25.11 (−33.26, −15.97)		2001–2011	−14.22 (−22.14, −5.49)
Venezuela	4	1959–1971	22.63 (12.43, 33.76)	4	1959–1971	40.54 (27.16, 55.33)
		1971–1980	−20.66 (−28.63, −11.8)		1971–1980	−18.23 (−27.21, −8.13)
		1980–1990	29.95 (20.90, 39.67)		1980–1988	36.17 (18.07, 57.05)
		1990–1993	−34.41 (−57.92, 2.22)		1988–1993	−10.96 (−23.12, 3.12)
		1993–2011	3.91 (2.12, 5.74)		1993–2011	2.22 (0.21, 4.27)

**Table 3:** APC in PvIRs and PfIRs (per 100,000 inhabitants) in the Americas, 1959–2011

Country	PvIR			PfIR		
	Number of joinpoints	Year range	APC (%; 95% confidence interval)	Number of joinpoints	Year range	APC (%; 95% confidence interval)
Argentina*	3	1959–1962	48.27 (−3.63, 128.11)			
		1962–1965	−53.24 (−88.33, 87.33)			
		1965–1996	1.38 (−1.04, 3.86)			
		1996–2011	−19.29 (−29.2, −8.00)			
Belize	5	1959–1972	−6.49 (−20.86, 10.49)	4	1959–1979	−20.72 (−27.41, −13.41)
		1972–1982	38.30 (21.53, 57.38)		1979–1983	166.18 (11.90, 533.16)
		1982–1990	−6.30 (−11.97, −0.25)		1983–1990	−28.45 (−41.49, −12.50)
		1990–1994	38.40 (17.06, 63.63)		1990–1995	44.52 (5.65, 97.69)
		1994–1999	−31.15 (−38.88, −22.45)		1995–2011	−30.70 (−41.79, −17.49)
		1999–2011	−14.25 (−20.52, −7.49)			
Bolivia	2	1959–1998	7.36 (6.15, 8.58)	3	1959–1990	2.31 (−0.36, 5.06)
		1998–2001	−31.03 (−56.57, 9.53)		1990–1998	23.54 (11.52, 36.86)
		2001–2011	−8.69 (−14.89, −2.03)		1998–2001	−49.70 (−70.98, −12.83)
					2001–2011	−6.89 (−19.10, 7.16)
Brazil	4	1960–1963	58.56 (−22.50, 224.44)	5	1960–1963	165.07 (−56.05, 1,498.76)
		1963–1970	−17.49 (−29.42, −3.55)		1963–1976	−4.49 (−8.32, −0.49)
		1970–1989	11.66 (9.39, 13.98)		1976–1988	17.21 (13.73, 20.80)
		1989–2006	−0.18 (−1.38, 1.04)		1988–2002	−10.89 (−12.54, −9.20)
		2006–2011	−11.06 (−17.98, −3.56)		2002–2005	21.96 (−30.02, 112.57)
					2005–2011	−23.18 (−30.20, −15.44)
Colombia	1	1959–1995	6.66 (5.47, 7.87)	1	1959–2002	3.07 (2.19, 3.97)
		1995–2011	−5.43 (−7.35, −3.48)		2002–2011	−14.93 (−21.08, −8.30)
Costa Rica	4	1959–1967	9.92 (−1.70, 22.90)	2	1959–1967	−48.27 (−60.18, −32.79)
		1967–1970	−64.33 (−86.86, −3.15)		1967–1976	60.50 (7.44, 139.76)
		1970–1989	5.04 (−3.79, 14.67)		1976–2011	−7.87 (−10.26, −5.42)
		1989–1992	97.86 (−5.00, 312.07)			
		1992–2011	−10.11 (−13.72, −6.34)			
Dominican Republic†				3	1959–1967	−41.15 (−55.42, −22.30)
					1967–1982	31.57 (18.90, 45.58)
					1982–1985	−45.09 (−77.19, 32.16)
					1985–2011	2.06 (−0.36, 4.54)
Ecuador	5	1959–1969	28.75 (11.16, 49.11)	3	1959–1993	5.48 (2.40, 8.63)
		1969–1973	−54.00 (−74.64, −16.56)		1993–1996	−51.45 (−80.00, 17.82)
		1973–1984	31.51 (15.79, 49.37)		1996–1999	170.85 (−4.70, 669.52)
		1984–1997	−10.84 (−16.59, −4.69)		1999–2011	−33.39 (−42.77, −22.47)
		1997–2001	41.76 (2.36, 96.33)			
		2001–2011	−33.35 (−43.61, −21.22)			
El Salvador	2	1959–1966	23.34 (5.83, 43.76)	5	1959–1963	−23.54 (−43.78, 3.98)
		1966–1982	0.87 (−1.86, 3.68)		1963–1966	78.10 (−40.59, 433.94)
		1982–2011	−22.94 (−27.43, −18.17)		1966–1969	−42.34 (−83.90, 106.45)
					1969–1975	35.04 (15.90, 57.34)
					1975–1984	−6.08 (−11.57, −0.24)
					1984–2011	−49.49 (−61.39, −33.93)
French Guiana	5	1960–1986	9.29 (4.83, 13.95)	2	1960–1994	9.27 (6.79, 11.80)
		1986–1990	82.59 (49.85, 122.49)		1994–2004	−7.25 (−11.66, −2.62)
		1990–1993	−47.29 (−61.88, −27.11)		2004–2011	−33.84 (−44.92, −20.54)
		1993–2003	−1.39 (−5.42, 2.81)			
		2003–2006	41.44 (−5.46, 111.60)			
		2006–2011	−33.10 (−39.22, −26.37)			
Guatemala	4	1959–1966	17.84 (−2.55, 42.50)	3	1959–1964	40.28 (6.70, 84.43)
		1966–1975	−15.12 (−26.77, −1.63)		1964–1973	−44.27 (−63.78, −14.24)
		1975–1978	124.65 (7.46, 369.63)		1973–1978	172.21 (0.19, 639.63)
		1978–2005	−4.75 (−5.97, −3.52)		1978–2011	−10.04 (−11.88, −8.15)
		2005–2011	−28.38 (−40.59, −13.66)			
Guyana	4	1959–1995	16.96 (14.42, 19.55)	4	1959–1984	4.89 (−4.63, 15.35)
		1995–1999	−18.52 (−36.77, 5.00)		1984–1987	165.52 (−16.33, 742.54)
		1999–2005	8.14 (−3.68, 21.41)		1987–1995	2.45 (−4.23, 9.60)
		2005–2008	−34.79 (−71.43, 48.84)		1995–2009	−8.06 (−11.22, −4.79)
		2008–2011	23.72 (−16.31, 82.91)		2009–2011	66.50 (−3.20, 186.39)
Haiti‡				3	1962–1983	10.50 (7.63, 13.45)
					1983–1986	−41.13 (−82.66, 99.90)
					1986–2004	−1.46 (−6.41, 3.76)
					2004–2011	21.75 (9.69, 35.13)
Honduras	1	1959–1996	1.52 (−0.08, 3.15)	5	1959–1964	−33.67 (−48.31, −14.87)
		1996–2011	−17.61 (−22.62, −12.28)		1964–1970	59.80 (24.75, 104.71)
					1970–1973	−60.34 (−89.34, 47.55)
					1973–1980	35.58 (14.36, 60.74)
					1980–1987	−25.87 (−37.06, −12.68)
					1987–2011	−1.94 (−4.56, 0.74)
Mexico	5	1959–1970	19.30 (9.50, 29.96)	5	1959–1967	−19.19 (−31.86, −4.17)
		1970–1978	−16.41 (−25.51, −6.20)		1967–1970	175.74 (−63.08, 1,959.58)
		1978–1985	33.31 (18.51, 49.96)		1970–1973	−50.62 (−77.09, 6.42)
		1985–1989	−8.18 (−22.48, 8.75)		1973–1985	10.55 (0.90, 21.13)
		1989–1992	−44.45 (−69.06, −0.27)		1985–1988	−50.86 (−83.90, 50.00)
		1992–2011	−11.12 (−17.22, −4.57)		1988–2011	−9.78 (−17.60, −1.23)
Nicaragua	2	1959–1992	1.27 (−0.46, 3.02)	0	1959–2011	−4.33 (−5.57, −3.08)
		1992–1996	22.56 (−4.63, 57.51)			
		1996–2011	−29.42 (−35.69, −22.53)			
Panama	4	1959–1983	−14.85 (−16.69, −12.96)	3	1959–1965	−21.45 (−43.60, 9.39)
		1983–1986	69.09 (−96.43, 7,909.03)		1965–1969	81.97 (−2.21, 238.61)
		1986–1997	−7.58 (−15.30, 0.84)		1969–1979	−33.98 (−42.17, −24.63)
		1997–2004	33.77 (18.25, 51.31)		1979–2011	5.47 (1.10, 10.03)
		2004–2011	−33.07 (−41.23, −23.77)			
Paraguay	3	1959–1967	55.60 (31.33, 84.36)	2	1959–1967	92.87 (57.41, 136.32)
		1967–1972	−56.42 (−74.09, −26.70)		1967–1973	−57.43 (−69.27, −41.02)
		1972–1999	6.46 (−3.05, 16.90)		1973–2011	−5.60 (−11.53, 0.73)
		1999–2011	−28.84 (−43.12, −10.98)			
Peru	2	1959–1991	5.09 (2.07, 8.19)	5	1959–1989	−8.34 (−11.52, −5.06)
		1991–1995	37.77 (10.14, 72.32)		1989–1994	264.76 (172.52, 388.23)
		1995–2011	−11.84 (−14.32, −9.29)		1994–1998	34.01 (27.09, 41.29)
					1998–2001	−41.51 (−49.21, −32.64)
					2001–2004	2.49 (−14.34, 22.63)
					2004–2011	−29.47 (−32.01, −26.84)
Suriname	3	1959–1988	12.48 (8.49, 16.62)	2	1959–1988	−0.19 (−3.33, 3.06)
		1988–1991	−58.29 (−98.56, 1,110.36)		1988–2001	13.94 (5.35, 23.24)
		1991–1998	75.14 (46.23, 109.76)		2001–2011	−28.84 (−39.98, −17.03)
		1998–2011	−9.12 (−11.33, −6.86)			
Venezuela	4	1959–1971	20.36 (8.29, 33.78)	5	1959–1972	33.63 (21.99, 46.38)
		1971–1980	−21.84 (−32.02, −10.13)		1972–1981	−25.25 (−32.71, −16.97)
		1980–1989	32.32 (17.93, 48.46)		1981–1988	58.54 (38.76, 81.14)
		1989–1993	−20.28 (−41.19, 8.06)		1988–1993	−23.54 (−32.98, −12.77)
		1993–2011	3.07 (0.75, 5.45)		1993–2002	−2.69 (−8.93, 3.99)
					2002–2011	10.80 (6.54, 15.23)

* Data from Argentina were not analyzed for PfIR.

† Data from Dominican Republic were not analyzed for PvIR.

‡ Data from Haiti were not analyzed for PvIR.

**Table 4:** AAPC in API and SPR by country, 2000–2011

Country	AAPC	
	API	SPR
Argentina	−19.26 (−29.14, −8.00)*	−2.67 (−8.23, 3.22)
Belize	−12.89 (−22.18, −2.49)*	−15.56 (−19.02, −11.95)*
Bolivia	−10.66 (−16.42, −4.51)*	−9.91 (−16.93, −2.29)*
Brazil	−6.86 (−10.75, −2.80)*	−7.23 (−11.93, −2.29)*
Colombia	−10.08 (−13.63, −6.39)*	−5.02 (−6.68, −3.33)*
Costa Rica	−9.82 (−13.53, −5.94)*	−21.18 (−38.82, 1.53)
Dominican Republic	2.00 (−0.49, 4.54)	3.25 (1.09, 5.46)*
Ecuador	−32.81 (−43.93, −19.49)*	−31.41 (−40.56, −20.85)*
El Salvador	−24.98 (−30.08, −19.51)*	−21.42 (−28.58, −13.54)*
French Guiana	−14.14 (−23.30, −3.88)*	−1.00 (−11.60, 10.90)*
Guatemala	−18.79 (−27.01, −9.66)*	−17.09 (−23.58, −10.06)*
Guyana	−0.76 (−11.32, 11.06)*	−1.01 (−3.68, 1.72)
Haiti	3.12 (1.02, 5.26)*	−5.268 (−6.44, 159.74)
Honduras	−17.86 (−22.83, −12.56)*	−9.63 (−14.46, −4.51)*
Mexico	−11.11 (−17.34, −4.41)*	−8.81 (−14.16, −3.13)*
Nicaragua	−28.46 (−34.53, −21.82)*	−27.44 (−33.18, −21.22)*
Panama	−13.07 (−27.28, 3.93)	−13.96 (−24.21, −2.33)*
Paraguay	0.73 (−5.36, 7.22)	2.67 (−3.11, 8.79)
Peru	−14.10 (−16.73, −11.38)*	−2.97 (−6.03, 0.19)
Suriname	−22.11 (−29.67, −13.73)*	−12.31 (−19.52, −4.46)*
Venezuela	3.91 (2.12, 5.74)*	2.22 (0.21, 4.27)*

* Significant at the 0.05 level.

**Table 5:** Last joinpoint year (API) and number of malaria cases by country

Country	Last joinpoint year	Number of malaria cases*	
		Joinpoint year	2011
Argentina	1996	2,048	18
Belize	2000	1,486	79
Bolivia	2001	15,765	7,143
Brazil	2006	549,469	267,045
Colombia	2001	231,233	64,436
Costa Rica	1992	6,951	17
Dominican Republic	1985	816	1,616
Ecuador	2001	108,903	1,233
El Salvador	1982	86,202	15
French Guiana	2009	3,462	1,209
Guatemala	2005	39,571	6,817
Guyana	2009	13,673	29,471
Haiti	2004	10,802	32,969
Honduras	1996	91,799	7,615
Mexico	1992	16,170	1,124
Nicaragua	1996	75,606	925
Panama	2004	5,095	354
Paraguay	1971	423	10
Peru	1996	211,561	22,878
Suriname	2001	16,003	795
Venezuela	1993	12,539	45,824

* Total cases include cases imported from other countries.
