# Supplementary material for: Malaria in the Americas: Trends from 1959 to 2011
Source: Am J Trop Med Hyg. 2015 Feb 4;92(2):302–16. doi: 10.4269/ajtmh.14-0368 (PMC4347333; doi:10.4269/ajtmh.14-0368)
Supplement: Supplementary file 1 [file SD2.pdf]

| Country | Year | | | | | | | | | | | | | | | | | | | | | | | | | | | | | | | | | | | | | | | | | | | | | | | | | | | |
|---|---|---|---|---|---|---|---|---|---|---|---|---|---|---|---|---|---|---|---|---|---|---|---|---|---|---|---|---|---|---|---|---|---|---|---|---|---|---|---|---|---|---|---|---|---|---|---|---|---|---|---|---|
| | 59 | 60 | 61 | 62 | 63 | 64 | 65 | 66 | 67 | 68 | 69 | 70 | 71 | 72 | 73 | 74 | 75 | 76 | 77 | 78 | 79 | 80 | 81 | 82 | 83 | 84 | 85 | 86 | 87 | 88 | 89 | 90 | 91 | 92 | 93 | 94 | 95 | 96 | 97 | 98 | 99 | 00 | 01 | 02 | 03 | 04 | 05 | 06 | 07 | 08 | 09 | 10 | 11 |
| Argentina | 1 | N | N | • | N | N | • | N | N | N | N | N | N | N | N | N | N | N | N | N | N | N | N | N | N | N | N | N | N | N | N | N | N | N | N | N | N | • | R | R | R | R | R | R | R | R | R | R | R | R | R | R | R |
| Belize | 1 | R | R | R | R | R | R | R | R | R | R | R | R | • | I | I | I | I | I | I | I | I | I | • | R | R | R | R | R | R | R | • | I | I | I | • | R | R | R | R | R | • | R | R | R | R | R | R | R | R | R | R | R |
| Bolivia | 1 | I | I | I | I | I | I | I | I | I | I | I | I | I | I | I | I | I | I | I | I | I | I | I | I | I | I | I | I | I | I | I | I | I | • | I | I | I | I | • | R | R | • | R | R | R | R | R | R | R | R | R | R |
| Brazil | … | 1 | N | N | • | R | R | R | R | R | R | R | R | R | R | • | I | I | I | I | I | I | I | I | I | I | I | I | • | R | R | R | R | R | R | R | R | R | R | R | R | R | R | R | R | R | R | • | R | R | R | R | R |
| Colombia | 1 | I | I | I | I | I | I | I | I | I | I | I | I | I | I | I | I | I | I | I | I | I | I | I | I | I | I | I | I | I | I | I | I | I | I | I | I | I | I | I | I | I | • | R | R | R | R | R | R | R | R | R | R |
| Costa Rica | 1 | N | N | N | N | N | N | N | • | N | N | • | N | N | N | N | N | N | N | N | N | N | N | N | N | N | N | N | N | N | • | N | N | • | R | R | R | R | R | R | R | R | R | R | R | R | R | R | R | R | R | R | R |
| Dominican Republic | 1 | R | R | R | R | R | R | R | R | • | I | I | I | I | I | I | I | I | I | I | I | I | I | • | N | N | • | N | N | N | N | N | N | N | N | N | N | N | N | N | N | N | N | N | N | N | N | N | N | N | N | N | N |
| Ecuador | 1 | I | I | I | I | I | I | I | I | I | • | R | R | R | R | • | I | I | I | I | I | I | I | I | I | • | R | R | R | R | R | R | R | R | R | R | R | • | N | N | N | N | • | R | R | R | R | R | R | R | R | R | R |
| El Salvador | 1 | I | I | I | I | I | I | I | I | I | I | I | I | I | I | I | I | I | I | I | I | I | I | • | R | R | R | R | R | R | R | R | R | R | R | R | R | R | R | R | R | R | R | R | R | R | R | R | R | R | R | R | R |
| French Guiana | 1 | I | I | I | I | I | I | I | I | I | I | I | I | I | I | I | I | I | I | I | I | I | I | I | I | I | I | • | N | N | • | R | R | R | R | R | R | R | R | R | R | R | R | R | R | R | R | R | R | • | N | N | N |
| Guatemala | 1 | N | N | N | N | N | N | • | R | R | R | R | R | R | R | R | • | I | I | • | R | R | R | R | R | R | R | R | R | R | R | R | R | R | R | R | R | R | R | R | R | R | R | R | R | R | • | R | R | R | R | R | R |
| Guyana | 1 | I | I | I | I | I | I | I | I | I | I | I | I | I | I | I | I | I | I | I | I | I | I | I | I | • | N | N | • | N | N | N | N | N | N | N | • | R | R | R | R | R | R | R | R | R | R | R | R | R | • | N | N |
| Haiti | … | … | … | 1 | I | I | I | I | I | I | I | I | I | I | I | I | I | I | I | I | I | I | I | I | • | N | N | N | • | N | N | N | N | N | N | N | … | N | N | N | N | N | N | … | … | • | I | I | I | I | I | I | I |
| Honduras | 1 | I | I | I | I | I | I | I | I | I | I | I | I | I | I | I | I | I | I | I | I | I | I | I | I | I | I | I | I | I | I | I | I | I | I | I | I | • | R | R | R | R | R | R | R | R | R | R | R | R | R | R | R |
| Mexico | 1 | I | I | I | I | I | I | I | I | I | I | • | R | R | R | R | R | R | R | • | I | I | I | I | I | I | • | N | N | N | • | R | R | • | R | R | R | R | R | R | R | R | R | R | R | R | R | R | R | R | R | R | R |
| Nicaragua | 1 | N | N | N | N | N | N | N | N | N | N | N | N | N | N | N | N | N | N | N | N | N | N | N | N | N | N | N | N | N | N | N | N | • | N | N | N | • | R | R | R | R | R | R | R | R | R | R | R | R | R | R | R |
| Panama | 1 | R | R | R | R | • | N | N | N | N | • | R | R | R | R | R | R | R | • | N | N | N | N | N | N | N | N | N | N | N | N | N | N | N | N | N | N | N | N | N | N | • | N | N | N | • | R | R | R | R | R | R | R |
| Paraguay | 1 | I | I | I | I | I | I | I | • | R | R | R | • | N | N | N | N | N | N | N | N | N | N | N | N | N | N | N | N | N | N | N | N | N | N | N | N | N | N | N | N | N | N | N | N | N | N | N | N | N | N | N | N |
| Peru | 1 | I | I | I | I | I | I | I | I | I | I | I | I | I | I | I | I | I | I | I | I | I | I | I | I | I | I | I | I | I | I | I | • | I | I | I | I | • | R | R | R | R | R | R | R | R | R | R | R | R | R | R | R |
| Suriname | 1 | N | N | N | N | N | N | N | N | N | N | N | N | N | N | N | N | N | N | N | N | N | N | N | N | N | N | N | N | N | • | I | I | I | I | I | I | I | I | I | I | I | • | R | R | R | R | R | R | R | R | R | R |
| Venezuela | 1 | I | I | I | I | I | I | I | I | I | I | I | • | R | R | R | R | R | R | R | R | • | I | I | I | I | I | I | I | I | I | • | N | N | • | I | I | I | I | I | I | I | I | I | I | I | I | I | I | I | I | I | I |
| Reduction | | 3 | 3 | 3 | 3 | 3 | 3 | 3 | 4 | 4 | 4 | 6 | 6 | 6 | 6 | 4 | 3 | 3 | 2 | 1 | 2 | 1 | 1 | 1 | 3 | 3 | 4 | 4 | 4 | 5 | 5 | 6 | 6 | 5 | 7 | 7 | 8 | 8 | 12 | 12 | 13 | 12 | 12 | 16 | 16 | 16 | 16 | 16 | 17 | 16 | 15 | 15 | 15 |
| Not Significant | | 5 | 6 | 5 | 5 | 5 | 5 | 5 | 4 | 5 | 4 | 3 | 4 | 5 | 5 | 5 | 5 | 5 | 5 | 6 | 6 | 6 | 6 | 6 | 7 | 8 | 8 | 10 | 9 | 11 | 7 | 8 | 9 | 7 | 7 | 7 | 5 | 4 | 5 | 5 | 5 | 4 | 4 | 3 | 3 | 2 | 2 | 2 | 2 | 2 | 3 | 4 | 4 |
| Increase | | 11 | 11 | 11 | 12 | 12 | 12 | 12 | 11 | 11 | 11 | 10 | 9 | 9 | 10 | 10 | 12 | 13 | 13 | 12 | 13 | 13 | 14 | 11 | 10 | 8 | 7 | 6 | 5 | 5 | 5 | 5 | 5 | 6 | 5 | 6 | 6 | 4 | 4 | 3 | 3 | 3 | 1 | 1 | 1 | 1 | 2 | 2 | 2 | 2 | 2 | 2 | 2 |

R- Reduction   I- Increase   N- No significant trend   … Missing Data   • Joinpoint   1 First year of series

Supplemental Table 1. Malaria trend (API) by year and country in the Americas and number of countries with type of trend, 1959–2011.

| Country | Year | | | | | | | | | | | | | | | | | | | | | | | | | | | | | | | | | | | | | | | | | | | | | | | | | | | |
|---|---|---|---|---|---|---|---|---|---|---|---|---|---|---|---|---|---|---|---|---|---|---|---|---|---|---|---|---|---|---|---|---|---|---|---|---|---|---|---|---|---|---|---|---|---|---|---|---|---|---|---|---|
| | 59 | 60 | 61 | 62 | 63 | 64 | 65 | 66 | 67 | 68 | 69 | 70 | 71 | 72 | 73 | 74 | 75 | 76 | 77 | 78 | 79 | 80 | 81 | 82 | 83 | 84 | 85 | 86 | 87 | 88 | 89 | 90 | 91 | 92 | 93 | 94 | 95 | 96 | 97 | 98 | 99 | 00 | 01 | 02 | 03 | 04 | 05 | 06 | 07 | 08 | 09 | 10 | 11 |
| Argentina | 1 | R | R | R | R | R | R | R | R | R | • | I | I | I | I | I | I | I | I | I | I | I | I | I | I | I | I | I | I | I | • | N | N | N | N | N | N | N | N | N | N | N | N | N | N | N | N | N | N | N | … | N | N |
| Belize | 1 | R | R | R | R | R | R | R | R | R | R | R | R | R | • | I | I | I | I | I | I | I | I | • | I | I | I | I | I | I | I | I | I | I | I | I | • | R | R | R | R | R | R | R | R | R | R | R | R | R | R | R | R |
| Bolivia | 1 | I | I | I | I | I | I | I | I | I | I | I | I | I | I | I | I | I | I | I | I | I | I | I | I | I | I | I | I | I | I | I | I | I | I | I | I | I | I | • | R | R | R | • | N | N | N | N | N | N | N | N | N |
| Brazil | … | 1 | R | R | R | R | R | R | R | R | R | R | R | R | R | • | I | I | I | I | I | I | I | I | I | I | I | I | • | I | I | I | I | I | I | I | I | I | I | I | • | N | N | • | N | N | • | R | R | R | R | R | R |
| Colombia | 1 | I | I | I | I | I | I | I | I | I | I | I | I | I | I | I | I | I | I | • | I | I | I | I | I | I | I | I | I | I | I | I | I | I | I | I | I | I | I | … | I | • | R | R | R | R | R | R | R | R | R | R | R |
| Costa Rica | 1 | R | R | R | • | N | N | N | • | R | R | • | I | I | I | I | I | I | I | I | I | I | I | I | I | I | I | I | I | I | I | I | I | I | I | I | I | I | I | I | I | I | I | I | I | I | • | R | R | R | R | R | R |
| Dominican Republic | 1 | N | • | R | R | R | R | R | • | I | I | I | I | I | I | I | I | I | I | I | I | I | I | • | N | N | • | I | I | I | I | I | I | I | I | I | I | I | I | I | I | I | I | I | I | I | I | I | I | I | I | I | I |
| Ecuador | 1 | R | R | R | R | R | • | N | N | • | R | R | R | R | R | R | R | R | R | R | R | • | I | I | I | • | N | N | N | N | N | N | N | N | N | N | N | N | N | N | N | N | N | • | R | R | R | R | R | R | R | R | R |
| El Salvador | 1 | I | I | I | I | I | I | I | I | I | I | I | I | I | I | I | I | I | I | I | I | I | I | I | I | • | R | R | R | R | R | R | R | R | R | R | R | R | R | R | R | R | R | R | R | R | R | R | R | R | R | R | R |
| French Guiana | … | 1 | I | I | I | I | I | I | I | I | I | I | I | I | I | I | I | I | I | I | I | I | I | I | I | I | I | I | I | I | • | N | N | • | N | N | N | N | N | … | N | N | N | N | N | N | • | N | N | • | R | R | R |
| Guatemala | 1 | R | R | R | R | R | R | R | R | R | R | R | R | R | R | R | • | I | I | • | N | N | N | N | N | N | N | • | I | I | I | I | I | I | I | I | I | I | I | … | I | I | I | I | I | I | • | R | R | R | R | R | R |
| Guyana | 1 | N | N | N | N | N | N | N | N | N | N | N | N | N | N | N | N | N | N | N | N | N | N | • | N | N | • | N | N | N | N | N | N | • | N | N | N | N | • | N | N | N | N | N | N | N | N | N | N | N | N | N | N |
| Haiti | … | … | … | 1 | I | I | I | I | I | I | I | I | I | I | I | I | I | I | I | I | I | I | I | I | I | I | I | I | I | I | I | I | I | I | I | I | I | I | I | I | I | I | I | I | … | • | N | N | N | N | N | N | N |
| Honduras | 1 | I | I | I | I | I | I | I | I | I | I | I | I | I | I | I | I | I | I | I | I | I | • | N | N | • | I | I | I | I | I | I | I | I | I | I | I | • | R | R | R | R | R | R | R | R | R | R | R | R | R | R | R |
| Mexico | 1 | I | I | I | I | I | I | I | I | I | I | • | R | R | R | R | R | R | R | • | I | I | I | I | I | I | • | N | N | N | • | R | R | • | R | R | R | R | R | R | R | R | R | R | R | R | R | R | R | R | R | R | R |
| Nicaragua | 1 | N | N | N | N | N | N | N | N | N | N | N | N | N | N | N | N | N | N | N | N | • | N | N | • | I | I | I | I | I | I | I | I | I | I | I | I | • | R | R | R | R | R | R | R | R | R | R | R | R | R | R | R |
| Panama | 1 | R | R | R | R | • | N | N | N | N | • | R | R | • | N | N | N | N | N | N | N | N | N | N | N | N | N | N | N | N | N | N | N | N | N | N | N | • | I | I | I | I | I | I | I | • | R | R | R | R | R | R | R |
| Paraguay | 1 | I | I | I | I | I | I | I | • | R | R | R | • | N | N | N | N | N | N | N | N | N | N | N | N | N | N | N | N | N | N | N | N | N | N | N | N | N | N | N | N | N | N | N | N | N | N | N | N | N | N | N | N |
| Peru | 1 | R | R | R | • | I | I | I | I | I | I | I | I | I | I | I | I | I | • | N | N | N | • | I | I | I | I | I | I | I | … | I | I | I | • | R | R | R | R | R | R | • | N | N | N | N | N | N | N | N | N | N | … |
| Suriname | 1 | N | N | N | N | N | N | N | N | N | N | N | N | N | N | N | N | N | • | I | I | I | I | I | I | I | I | I | I | I | I | I | I | I | I | I | I | I | I | I | I | I | • | R | R | R | R | R | R | R | R | R | R |
| Venezuela | 1 | I | I | I | I | I | I | I | I | I | I | I | • | R | R | R | R | R | R | R | R | • | I | I | I | I | I | I | I | • | N | N | N | N | • | I | I | I | I | I | I | I | I | I | I | I | I | I | I | I | I | I | I |
| Reduction | | 7 | 8 | 9 | 7 | 6 | 5 | 5 | 4 | 6 | 6 | 6 | 6 | 6 | 5 | 4 | 3 | 3 | 3 | 2 | 2 | 0 | 0 | 0 | 0 | 0 | 1 | 1 | 1 | 1 | 1 | 2 | 2 | 1 | 2 | 3 | 3 | 4 | 6 | 6 | 7 | 6 | 7 | 7 | 8 | 8 | 9 | 12 | 12 | 12 | 13 | 13 | 13 |
| Not Significant | | 4 | 3 | 3 | 3 | 4 | 5 | 6 | 5 | 4 | 3 | 3 | 3 | 4 | 5 | 5 | 5 | 5 | 4 | 5 | 6 | 5 | 5 | 5 | 6 | 5 | 4 | 5 | 5 | 5 | 5 | 7 | 7 | 5 | 6 | 6 | 6 | 5 | 4 | 4 | 5 | 6 | 7 | 5 | 7 | 7 | 6 | 7 | 7 | 6 | 5 | 6 | 5 |
| Increase | | 7 | 8 | 8 | 9 | 10 | 10 | 10 | 9 | 10 | 10 | 10 | 10 | 10 | 10 | 11 | 12 | 13 | 12 | 11 | 13 | 13 | 14 | 13 | 14 | 13 | 13 | 14 | 14 | 14 | 11 | 12 | 12 | 12 | 11 | 12 | 11 | 9 | 10 | 7 | 8 | 7 | 6 | 6 | 5 | 4 | 2 | 2 | 2 | 2 | 2 | 2 | 2 |

R- Reduction   I- Increase   N- No significant trend   … Missing Data   • Joinpoint   1 First year of series

Supplemental Table 2. Slide positivity rate trend for malaria by year and country in the Americas and number of countries with type of trend, 1959–2011.

| Country | 59 | 60 | 61 | 62 | 63 | 64 | 65 | 66 | 67 | 68 | 69 | 70 | 71 | 72 | 73 | 74 | 75 | 76 | 77 | 78 | 79 |
|---|---|---|---|---|---|---|---|---|---|---|---|---|---|---|---|---|---|---|---|---|---|
| Argentina | 1 | N | N | • | N | N | • | N | N | N | N | N | N | N | N | N | N | N | N | N | N |
| Belize | 1 | N | N | N | N | N | N | N | N | N | N | N | N | • | I | I | I | I | I | I | I |
| Bolivia | 1 | I | I | I | I | I | I | I | I | I | I | I | I | I | I | I | I | I | I | I | I |
| Brazil | … | 1 | N | N | • | R | R | R | R | R | R | • | I | I | I | I | I | I | I | I | I |
| Colombia | 1 | I | I | I | I | I | I | I | I | I | I | I | I | I | I | I | I | I | I | I | I |
| Costa Rica | 1 | N | N | N | N | N | N | N | • | R | R | • | N | N | N | N | N | N | N | N | N |
| Ecuador | 1 | I | I | I | I | I | I | I | I | I | • | R | R | R | • | I | I | I | I | I | I |
| El Salvador | 1 | I | I | I | I | I | I | • | N | N | N | N | N | N | N | N | N | N | N | N | N |
| French Guiana | … | 1 | I | I | I | I | I | I | I | I | I | I | I | I | I | I | I | I | I | I | I |
| Guatemala | 1 | N | N | N | N | N | N | • | R | R | R | R | R | R | R | R | • | I | I | • | R |
| Guyana | 1 | I | I | I | I | I | I | I | I | I | I | I | I | I | I | I | I | I | I | I | I |
| Honduras | 1 | N | N | N | N | N | N | N | N | N | N | N | N | N | N | N | N | N | N | N | N |
| Mexico | 1 | I | I | I | I | I | I | I | I | I | I | • | R | R | R | R | R | R | R | • | I |
| Nicaragua | 1 | N | N | N | N | N | N | N | N | N | N | N | N | N | N | N | N | N | N | N | N |
| Panama | 1 | R | R | R | R | R | R | R | R | R | R | R | R | R | R | R | R | R | R | R | R |
| Paraguay | 1 | I | I | I | I | I | I | I | • | R | R | R | R | • | N | N | N | N | N | N | N |
| Peru | 1 | I | I | I | I | I | I | I | I | I | I | I | I | I | I | I | I | I | I | I | I |
| Suriname | 1 | I | I | I | I | I | I | I | I | I | I | I | I | I | I | I | I | I | I | I | I |
| Venezuela | 1 | I | I | I | I | I | I | I | I | I | I | I | • | R | R | R | R | R | R | R | R |
| Reduction | | 1 | 1 | 1 | 1 | 2 | 2 | 2 | 3 | 5 | 5 | 4 | 5 | 5 | 4 | 4 | 3 | 3 | 3 | 2 | 3 |
| Not Significant | | 6 | 7 | 6 | 6 | 6 | 5 | 5 | 5 | 5 | 5 | 5 | 6 | 5 | 6 | 6 | 6 | 6 | 6 | 6 | 6 |
| Increase | | 10 | 11 | 11 | 11 | 11 | 11 | 10 | 9 | 9 | 8 | 7 | 7 | 7 | 8 | 9 | 9 | 10 | 10 | 9 | 10 |

| Country | 80 | 81 | 82 | 83 | 84 | 85 | 86 | 87 | 88 | 89 | 90 | 91 | 92 | 93 | 94 | 95 | 96 | 97 | 98 | 99 | 00 | 01 | 02 | 03 | 04 | 05 | 06 | 07 | 08 | 09 | 10 | 11 |
|---|---|---|---|---|---|---|---|---|---|---|---|---|---|---|---|---|---|---|---|---|---|---|---|---|---|---|---|---|---|---|---|---|
| Argentina | N | N | N | N | N | N | N | N | N | N | N | N | N | N | N | N | • | R | R | R | R | R | R | R | R | R | R | R | R | R | R | R |
| Belize | I | I | • | R | R | R | R | R | R | R | • | I | I | I | • | R | R | R | R | • | R | R | R | R | R | R | R | R | R | R | R | R |
| Bolivia | I | I | I | I | I | I | I | I | I | I | I | I | I | I | I | I | I | I | • | N | N | • | R | R | R | R | R | R | R | R | R | R |
| Brazil | I | I | I | I | I | I | I | I | I | • | N | N | N | N | N | N | N | N | N | N | N | N | N | N | N | N | • | R | R | R | R | R |
| Colombia | I | I | I | I | I | I | I | I | I | I | I | I | I | I | I | • | R | R | R | R | R | R | R | R | R | R | R | R | R | R | R | R |
| Costa Rica | N | N | N | N | N | N | N | N | N | • | N | N | • | R | R | R | R | R | R | R | R | R | R | R | R | R | R | R | R | R | R | R |
| Ecuador | I | I | I | I | • | R | R | R | R | R | R | R | R | R | R | R | R | • | I | I | I | • | R | R | R | R | R | R | R | R | R | R |
| El Salvador | N | N | • | R | R | R | R | R | R | R | R | R | R | R | R | R | R | R | R | R | R | R | R | R | R | R | R | R | R | R | R | R |
| French Guiana | I | I | I | I | I | I | • | I | I | I | • | R | R | • | N | N | N | N | N | N | N | N | N | • | N | N | • | R | R | R | R | R |
| Guatemala | R | R | R | R | R | R | R | R | R | R | R | R | R | R | R | R | R | R | R | R | R | R | R | R | R | • | R | R | R | R | R | R |
| Guyana | I | I | I | I | I | I | I | I | I | I | I | I | I | I | I | • | N | N | N | • | N | N | N | N | N | • | N | N | • | N | N | N |
| Honduras | N | N | N | N | N | N | N | N | N | N | N | N | N | N | N | N | • | R | R | R | R | R | R | R | R | R | R | R | R | R | R | R |
| Mexico | I | I | I | I | I | • | N | N | N | • | R | R | • | R | R | R | R | R | R | R | R | R | R | R | R | R | R | R | R | R | R | R |
| Nicaragua | N | N | N | N | N | N | N | N | N | N | N | N | • | N | N | N | • | R | R | R | R | R | R | R | R | R | R | R | R | R | R | R |
| Panama | R | R | R | • | N | N | • | N | N | N | N | N | N | N | N | N | N | • | I | I | I | I | I | I | • | R | R | R | R | R | R | R |
| Paraguay | N | N | N | N | N | N | N | N | N | N | N | N | N | N | N | N | N | N | N | • | R | R | R | R | R | R | R | R | R | R | R | R |
| Peru | I | I | I | I | I | I | I | I | I | I | I | • | I | I | I | • | R | R | R | R | R | R | R | R | R | R | R | R | R | R | R | R |
| Suriname | I | I | I | I | I | I | I | I | • | N | N | • | I | I | I | I | I | I | • | R | R | R | R | R | R | R | R | R | R | R | R | R |
| Venezuela | • | I | I | I | I | I | I | I | I | • | N | N | N | • | I | I | I | I | I | I | I | I | I | I | I | I | I | I | I | I | I | I |
| Reduction | 2 | 2 | 2 | 3 | 3 | 4 | 4 | 4 | 4 | 4 | 4 | 5 | 4 | 5 | 5 | 6 | 8 | 10 | 10 | 10 | 12 | 12 | 14 | 14 | 14 | 14 | 15 | 17 | 17 | 17 | 17 | 17 |
| Not Significant | 6 | 6 | 5 | 5 | 6 | 6 | 6 | 7 | 7 | 6 | 9 | 8 | 6 | 6 | 7 | 7 | 5 | 4 | 4 | 3 | 4 | 3 | 3 | 2 | 3 | 2 | 1 | 1 | 0 | 1 | 1 | 1 |
| Increase | 10 | 11 | 10 | 10 | 9 | 8 | 7 | 8 | 7 | 5 | 4 | 4 | 6 | 6 | 6 | 3 | 3 | 3 | 3 | 3 | 3 | 2 | 2 | 2 | 1 | 1 | 1 | 1 | 1 | 1 | 1 | 1 |

R- Reduction I- Increase N- No significant trend … Missing Data • Joint point year 1 First year of series

Supplemental Table 3. *Plasmodium vivax* incidence trend for malaria by year and country in the Americas and number of countries with type of trend, 1959–2011.

| Country | 59 | 60 | 61 | 62 | 63 | 64 | 65 | 66 | 67 | 68 | 69 | 70 | 71 | 72 | 73 | 74 | 75 | 76 | 77 | 78 | 79 |
|---|---|---|---|---|---|---|---|---|---|---|---|---|---|---|---|---|---|---|---|---|---|
| Belize | 1 | R | R | R | R | R | R | R | R | R | R | R | R | R | R | R | R | R | R | R | • |
| Bolivia | 1 | N | N | N | N | N | N | N | N | N | N | N | N | N | N | N | N | N | N | N | N |
| Brazil | … | 1 | N | N | • | R | R | R | R | R | R | R | R | R | R | R | R | • | I | I | I |
| Colombia | 1 | I | I | I | I | I | I | I | I | I | I | I | I | I | I | I | I | I | I | I | I |
| Costa Rica | 1 | R | R | R | R | R | R | R | • | I | I | I | I | I | I | I | I | • | R | R | R |
| Dominican Republic | 1 | R | R | R | R | R | R | R | • | I | I | I | I | I | I | I | I | I | I | I | I |
| Ecuador | 1 | I | I | I | I | I | I | I | I | I | I | I | I | I | I | I | I | I | I | I | I |
| El Salvador | 1 | N | N | N | • | N | N | • | N | N | • | I | I | I | I | I | • | R | R | R | R |
| French Guiana | … | 1 | I | I | I | I | I | I | I | I | I | I | I | I | I | I | I | I | I | I | I |
| Guatemala | 1 | I | I | I | I | • | R | R | R | R | R | R | R | R | • | I | I | I | I | • | R |
| Guyana | 1 | N | N | N | N | N | N | N | N | N | N | N | N | N | N | N | N | N | N | N | N |
| Haiti | … | … | … | 1 | I | I | I | I | I | I | I | I | I | I | I | I | I | I | I | I | I |
| Honduras | 1 | R | R | R | R | • | I | I | I | I | I | • | N | N | • | I | I | I | I | I | I |
| Mexico | 1 | R | R | R | R | R | R | R | • | N | N | • | N | N | • | I | I | I | I | I | I |
| Nicaragua | 1 | R | R | R | R | R | R | R | R | R | R | R | R | R | R | R | R | R | R | R | R |
| Panama | 1 | N | N | N | N | N | • | N | N | N | • | R | R | R | R | R | R | R | R | R | • |
| Paraguay | 1 | I | I | I | I | I | I | I | • | R | R | R | R | R | • | N | N | N | N | N | N |
| Peru | 1 | R | R | R | R | R | R | R | R | R | R | R | R | R | R | R | R | R | R | R | R |
| Suriname | 1 | N | N | N | N | N | N | N | N | N | N | N | N | N | N | N | N | N | N | N | N |
| Venezuela | 1 | I | I | I | I | I | I | I | I | I | I | I | I | • | R | R | R | R | R | R | R |
| Reduction | | 7 | 7 | 7 | 7 | 7 | 8 | 8 | 5 | 6 | 6 | 7 | 7 | 7 | 6 | 6 | 6 | 6 | 7 | 7 | 6 |
| Not Significant | | 5 | 6 | 6 | 4 | 5 | 4 | 4 | 5 | 6 | 4 | 3 | 5 | 5 | 3 | 4 | 4 | 4 | 4 | 4 | 4 |
| Increase | | 5 | 6 | 6 | 7 | 6 | 7 | 7 | 6 | 8 | 8 | 8 | 8 | 7 | 7 | 10 | 9 | 8 | 9 | 8 | 8 |

| Country | 80 | 81 | 82 | 83 | 84 | 85 | 86 | 87 | 88 | 89 | 90 | 91 | 92 | 93 | 94 | 95 | 96 | 97 | 98 | 99 | 00 | 01 | 02 | 03 | 04 | 05 | 06 | 07 | 08 | 09 | 10 | 11 |
|---|---|---|---|---|---|---|---|---|---|---|---|---|---|---|---|---|---|---|---|---|---|---|---|---|---|---|---|---|---|---|---|---|
| Belize | I | I | I | • | R | R | R | R | R | R | • | I | I | I | I | • | R | R | R | R | R | R | R | R | R | R | R | R | R | R | R | R |
| Bolivia | N | N | N | N | N | N | N | N | N | N | • | I | I | I | I | I | I | I | • | R | R | • | N | N | N | N | N | N | N | N | N | N |
| Brazil | I | I | I | I | I | I | I | I | • | R | R | R | R | R | R | R | R | R | R | R | R | R | • | N | N | • | R | R | R | R | R | R |
| Colombia | I | I | I | I | I | I | I | I | I | I | I | I | I | I | I | I | I | I | I | I | I | I | • | R | R | R | R | R | R | R | R | R |
| Costa Rica | R | R | R | R | R | R | R | R | R | R | R | R | R | R | R | R | R | R | R | R | R | R | R | R | R | R | R | R | R | R | R | R |
| Dominican Republic | I | I | • | N | N | • | N | N | N | N | N | N | N | N | N | N | N | N | N | N | N | N | N | N | N | N | N | N | N | N | N | N |
| Ecuador | I | I | I | I | I | I | I | I | I | I | I | I | I | • | N | N | • | N | N | • | R | R | R | R | R | R | R | R | R | R | R | R |
| El Salvador | R | R | R | R | • | R | R | R | R | R | R | R | R | R | R | R | R | R | R | R | R | R | R | R | R | R | R | R | R | R | R | R |
| French Guiana | I | I | I | I | I | I | I | I | I | I | I | I | I | I | • | R | R | R | R | R | R | R | R | R | • | R | R | R | R | R | R | R |
| Guatemala | R | R | R | R | R | R | R | R | R | R | R | R | R | R | R | R | R | R | R | R | R | R | R | R | R | R | R | R | R | R | R | R |
| Guyana | N | N | N | N | • | N | N | • | N | N | N | N | N | N | N | • | R | R | R | R | R | R | R | R | R | R | R | R | R | • | N | N |
| Haiti | I | I | I | • | N | N | • | N | N | N | N | N | N | N | N | … | N | N | N | N | N | N | … | … | • | I | I | I | I | I | I | I |
| Honduras | • | R | R | R | R | R | R | • | N | N | N | N | N | N | N | N | N | N | N | N | N | N | N | N | N | N | N | N | N | N | N | N |
| Mexico | I | I | I | I | I | • | N | N | • | R | R | R | R | R | R | R | R | R | R | R | R | R | R | R | R | R | R | R | R | R | R | R |
| Nicaragua | R | R | R | R | R | R | R | R | R | R | R | R | R | R | R | R | R | R | R | R | R | R | R | R | R | R | R | R | R | R | R | R |
| Panama | I | I | I | I | I | I | I | I | I | I | I | I | I | I | I | I | I | I | I | I | I | I | I | I | I | I | I | I | I | I | I | I |
| Paraguay | N | N | N | N | N | N | N | N | N | N | N | N | N | N | N | N | N | N | N | N | N | N | N | N | N | N | N | N | N | N | N | N |
| Peru | R | R | R | R | R | R | R | R | R | • | I | I | I | I | • | I | I | I | • | R | R | • | N | N | • | R | R | R | R | R | R | R |
| Suriname | N | N | N | N | N | N | N | N | • | I | I | I | I | I | I | I | I | I | I | I | I | • | R | R | R | R | R | R | R | R | R | R |
| Venezuela | R | • | I | I | I | I | I | I | • | R | R | R | R | • | N | N | N | N | N | N | N | N | • | I | I | I | I | I | I | I | I | I |
| Reduction | 6 | 6 | 6 | 6 | 6 | 7 | 7 | 6 | 6 | 8 | 7 | 7 | 7 | 6 | 6 | 7 | 9 | 9 | 9 | 11 | 12 | 10 | 10 | 11 | 10 | 12 | 13 | 13 | 13 | 12 | 12 | 12 |
| Not Significant | 4 | 4 | 4 | 5 | 5 | 5 | 6 | 6 | 6 | 6 | 5 | 5 | 5 | 5 | 7 | 5 | 5 | 6 | 6 | 5 | 5 | 5 | 5 | 6 | 5 | 4 | 4 | 4 | 4 | 4 | 5 | 5 |
| Increase | 9 | 9 | 9 | 7 | 7 | 6 | 6 | 6 | 4 | 5 | 6 | 8 | 8 | 7 | 5 | 5 | 5 | 5 | 3 | 3 | 3 | 2 | 1 | 2 | 2 | 3 | 3 | 3 | 3 | 3 | 3 | 3 |

R- Reduction I- Increase N- No significant trend … Missing Data 1 First year of series

Supplemental Table 4. *Plasmodium falciparum* incidence trend for malaria by year and country in the Americas and number of countries with type of trend, 1959–2011.
